# Correlation between the use of social media and the self-esteem of adults with autism in their workplace

**DOI:** 10.1192/j.eurpsy.2025.1607

**Published:** 2025-08-26

**Authors:** M. M. N. Matsumoto, B. F. A. da Silva, D. R. Molini-Avejonas

**Affiliations:** 1 University of Sao Paulo, Sao Paulo, Brazil

## Abstract

**Introduction:**

There is a greater inequality in employment and underemployment among adults with Autism Spectrum Disorder (ASD) compared to their peers. Aspects such as sustained eye contact, interpreting non-verbal cues, understanding non-literal language, exhibiting cognitive inflexibility, and limitations in interpreting others’ perspectives impact their communication. Due to these difficulties, they are often subject to social embarrassment, isolation, and insecurity when initiating conversations. Considering that contemporary interactions have intensified through social media, these networks can be a facilitator of social inclusion, especially as they are digital environments—structured, free from unexpected stimuli, offering additional processing time, and without the need to interpret prosody and intonation. In this context, to understand its impact in the workplace for this population, one of the factors to consider is the self-esteem of these individuals within the workplace, given that self-esteem is a fundamental indicator of self-worth and self-acceptance, impacting mental health.

**Objectives:**

To verify the correlation between social media use and self-esteem in adults with ASD in their workplace.

**Methods:**

This is a prospective, qualitative-quantitative study based on the Ethics Committee for Research number 65890317.9.0000.0065. Data were collected via an electronic form. Questionnaires: personal/social questions prepared by the authors; adapted protocols: Rosenberg Self-Esteem Scale and Facebook Intensity Scale.

**Results:**

A total of 132 adults with self-reported ASD, 68% of whom had ASD with comorbidities. Regarding gender and sexual orientation, 66% were cisgender and heterosexual women, and 62% were cisgender and heterosexual men. Concerning remuneration and education, 44.7% had completed higher education, earning between two to three thousand reais per month. Additionally, 61.4% reported not having inclusive strategies in their workplace. The overall correlation between self-esteem and social media use at work showed that 65.9% use social media moderately, of which 12.9%, 18.9%, and 34.1% have low, high, and medium self-esteem, respectively. Correlating personal/social questions with self-esteem, 34.8% never feel comfortable with group conversations, of which 11.4% have low self-esteem (p-value 0.008), 48.5% feel distressed at work (p-value 0.06), 62.9% are excessively concerned about work (p-value 0.02), and 49.2% find it difficult to assert themselves at work (p-value 0.02). (Image 1)

**Image 1:**

**Figure:**
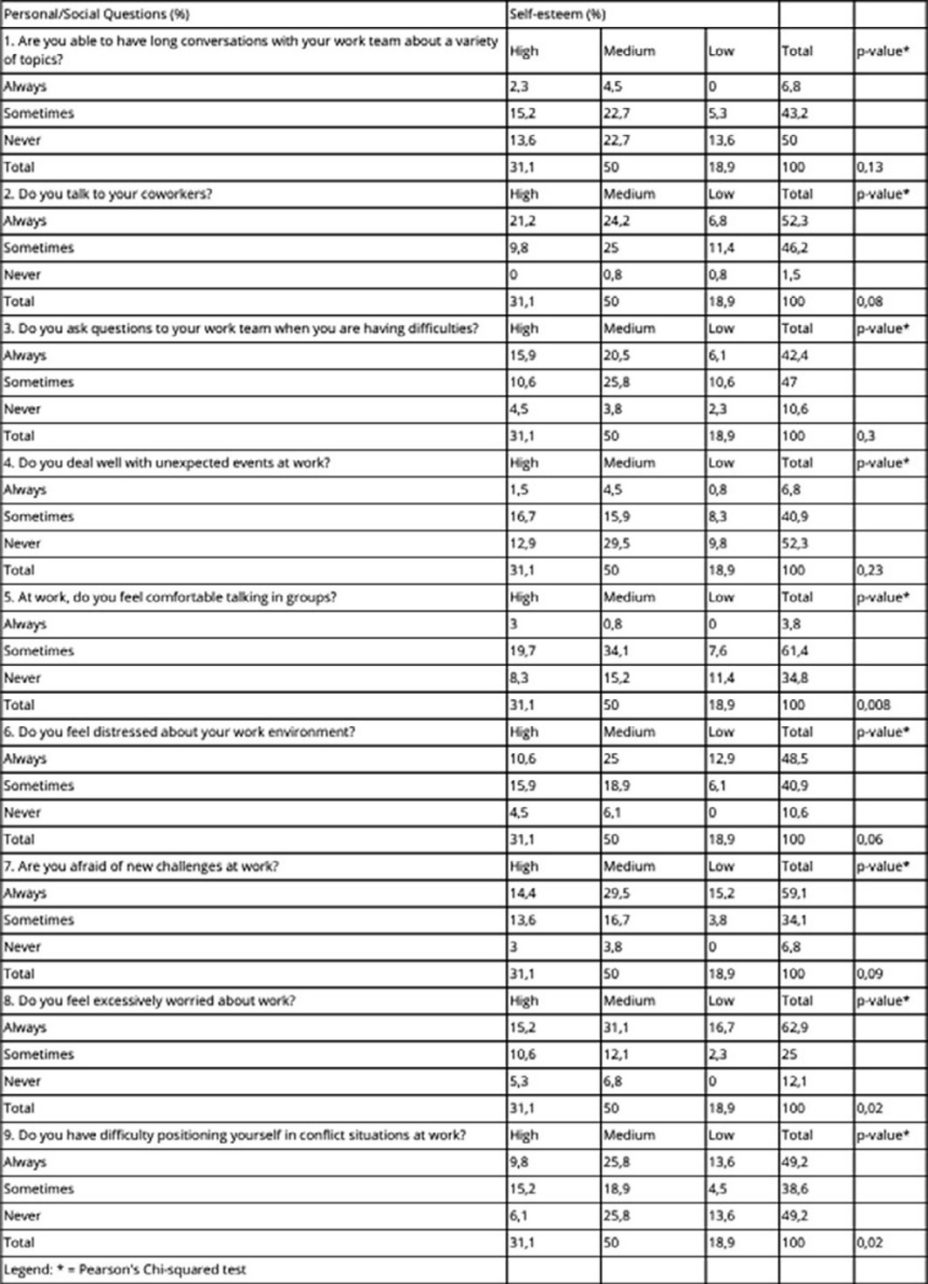

**Conclusions:**

This study did not statistically demonstrate a correlation between social media use and self-esteem in the workplace. However, the challenges encountered in the workplace, such as the lack of inclusive strategies, social difficulties that generate fear, distress, and worry, corroborate the literature regarding the vulnerability this population is exposed to.

**Disclosure of Interest:**

None Declared

